# A Retrospective Study of Metastatic Renal Cell Carcinoma Patients With Brain Metastases

**DOI:** 10.7759/cureus.34014

**Published:** 2023-01-20

**Authors:** Martin Zapata Laguado, Heliberto Paez Quintero, Andres Gomez, Rodolfo Varela

**Affiliations:** 1 Clinical Oncology, Universidad el Bosque, Bogotá, COL; 2 Oncology, Universidad el Bosque, Bogotá, COL; 3 Clinical Oncology, Universidad Militar Nueva Granada, Instituto Nacional de Cancerologia, Bogotá, COL; 4 Urology, Instituto Nacional de Cancerología, Bogotá, COL

**Keywords:** clear, brain, kidney, metastastic, cancer

## Abstract

Abstract

Metastatic renal cell carcinoma (mRCC) involving the central nervous system is currently an excluded subgroup of patients in the systemic treatment; for this reason, there is no solid data to support the efficacy of therapies in this subgroup. That is why it is important to describe real-life experiences in order to know if there is a special change in clinical behavior or treatment response in these kinds of patients.

Patients and methods

A retrospective review was performed to characterize mRCC patients diagnosed with brain metastases (BrM) during treatment at the National Institute of Cancerology of Bogota, Colombia. Descriptive statistics and time-to-event methods are used to evaluate the cohort. For the descriptive measures of quantitative variables, the mean with standard deviation was taken, and the minimum and maximum values were reported. In the case of qualitative variables, absolute and relative frequencies were used. The software used was R - Project v4.1.2 (R Foundation for Statistical Computing, Vienna, Austria).

Results

A total of 16 patients were included with mRCC between January 2017 to August 2022 with a median 35.1-month follow-up, 4/16 (25%) were diagnosed with BrM at the time of screening and 12/16 (75%) during treatment. The International Metastatic RCC Database Consortium risk (IMDC) was favorable for 12.5%, intermediate for 43.7%, and poor for 25%, and not classified for 18.8%, BrM involvement was multifocal in 50% of the population and localized, brain-directed therapy was done in 43.7% of patients, predominantly palliative radiotherapy. Median overall survival (OS) for all the patients regardless of the time of metastatic presentation of the disease in the central nervous system was 53.5 months (0-70.3), and OS for cases with central nervous system involvement was 10.9 months. IMDC risk did not correlate with survival (log-rank, p=0.67). The OS for the subgroup of patients who debut with metastatic disease in the central nervous system is different from the group that developed metastasis in the progression of their disease (OS of 42 vs 3.6 months, respectively).

Conclusions

This is the largest descriptive study in Latin America and the second in the world from one institution that admits patients with metastasic renal cell carcinoma and central nervous system metastasis. In these kinds of patients with metastatic disease or progression to the central nervous system, there is a hypothesis that shows more aggressive clinical behavior. There is limited data on locoregional intervention to metastatic disease in the nervous system drastically, but trends show this could impact overall survival outcomes.

## Introduction

In 2020, there were a total of 431,288 new cases of kidney cancer (KC) worldwide [1]. Histologically, renal cell carcinoma (RCC) represents the vast majority (90%) of KC cases, which predominantly include clear cell RCC (ccRCC; 70%) [2]. Histologic subtypes differ in clinical features, outcomes, and genetic determinants.

Brain metastases (BrM) are a presentation of the spectrum of disseminated disease with a cumulative incidence at five years as seen in previous studies (between 7-9.8% at five years) [3,4].

Currently, first-line phase 3 studies of metastatic disease that include immunotherapy and/or therapy with IL-2-inducible tyrosine kinase (ITK) inhibitors have excluded disseminated disease with involvement in the central nervous system in the first line, for example, CheckMate 214, which investigated the combination of nivolumab plus ipilimumab versus sunitinib, excluded all patients with central nervous system (CNS) metastases [5], whereas Keynote 426 (pembrolizumab/axitinib) [6], Javelin 101 (avelumab/axitinib) [7], and CheckMate 9ER (nivolumab/cabozantinib) [8] excluded patients with symptomatic or active CNS metastases. For this reason, there are no published data that support the efficacy of therapies in this subgroup of patients.

Additionally, the GETUG-AFU 26 NIVOREN study [9], a phase 2 study to evaluate the safety and efficacy of nivolumab monotherapy in patients who progressed to TKI inhibitors included 73 patients with documented BrM and were divided into two arms: 39 patients who did not receive local therapy, and 34 patients who received local management, showing a response rate of 12% in the first cohort, which emphasizes the importance of local therapy before systemic therapy.

CheckMate 920 is a phase 3b/4 study that evaluated the safety and efficacy of double immunotherapy (ipilimumab and nivolumab) on previously untreated patients with central nervous system involvement of renal cancer. After a minimum follow-up of 24.5 months (n = 28), the objective response rate was 32% (95% CI, 14.9%-53.5%) with a median duration of response of 24.0 months; the median progression-free survival was 9.0 months (95% CI, 2.9-12.0 months), and the median overall survival was not reached (95% CI, 14.1 months to present) at the time of the study's publication in 2022.

A study of 16 patients in our institution was carried out to describe the treatment and follow-up of the population of interest.

## Materials and methods

Patients and methods

After obtaining approval with number 760616 from the ethics committee of the Instituto Nacional de Cancerologia in Bogota, Colombia, a retrospective record review was performed. A pooled anonymous database of selected mCRC patients who had imaging or documented pathologic diagnosis in the central nervous system with confirmed primary renal cancer was constructed.

Patients with suspected second primary renal cancer or without images documenting the disease in the central nervous system were excluded. Not all enrolled patients presented with primary metastatic disease in the central nervous system; they may have presented with metastasis at another healthcare facility and have been admitted to our institution due to disease progression in the central nervous system. Baseline clinical characteristics collected included demographic data and their International Metastatic RCC Database Consortium (IMDC) risk status. The brain metastases were characterized by the number; the size of the metastases was not differentiated, nor was their location.

Patients were managed according to best practices at the time of presentation associated with international guidelines, and therapeutic interventions and specific outcomes were recorded.

Between January 2017 and August 2022, 16 patients were admitted with a diagnosis of mRCC with central nervous system involvement. Continuous variables were summarized using the median with standard deviation, while categorical data were tabulated using frequencies and percentages. Median overall survival (OS) and 95% CI were calculated using the Kaplan-Meier method. Overall survival was measured from diagnosis of brain metastases to death from any cause, and patients were excluded at the time of the last follow-up if they were alive or without follow-up data. The log-rank test was used to compare survival results by clinical variables. For statistical analysis, R - Project v4.1.2 was used (R Foundation for Statistical Computing, Vienna, Austria).

## Results

In total the admitted patients were 16, with a diagnosis of mRCC with central nervous system involvement. A total of 1223 patients were reviewed; only 22 patients had involvement in the central nervous system. Of these, six patients were not admitted due to having either confirmed or suspected second primary renal cancer, of which 16 patients were eligible to participate.

The characteristics of the patients are summarized in Table 1. All the admitted patients had histology of clear cell renal cancer, males were 62.5% (n=10), with a mean age of 61 years (SD 10.3 years with a minimum age of 36 and a maximum of 78 years). Of the 16 patients in the case series, four patients (25%) debuted with the disease in the CNS at the time of diagnosis, the remaining 12 patients (75%) developed disease with CNS involvement during the progression of their treatment. Of these 12 patients, seven patients (58%) had the disease in the second line of treatment. The remaining five patients progressed further; four (33%) were diagnosed in the third line of treatment and one (9%) in the fourth line. No patient was asymptomatic at the time of screening; 100% presented symptoms in the central nervous system, which led to neuroimaging. Around 68.7% (n=11) of the patient's ECOG status at diagnosis was 0-1, with the remaining 31.2% measuring ECOG 2; a nephrectomy was performed at some point during the treatment in 68.7% (n=11).

**Table 1:** Characteristics of the general population ITK: Tyrosine kinase inhibitor

Variable	N: 16 (100%)
Gender	
Male	10 (62.5%)
Female	6(37.5%)
Age (years)	61 (36; 78)
ECOG (diagnosis)	
0	3 (18.7%)
1	8 (50%)
2	5 (31.2%
Time of diagnosis of the disease in the central nervous system	
Initial	4 (25%)
Progression	12 (75%)
Number of lesions in the central nervous system	
1 lesion	8 (50%)
More than 1	8 (50%)
IMDC (International Metastatic RCC Database Consortium)	
Favorable	2 (12.5%)
Intermediate	7 (43.7%)
Poor	4 (25%)
Unknown	3 (18.8%)
Nephrectomy	
Yes	11 (68.7%)
Total	10 (90%)
Partial	1 (10%)
No	5 (31.2%)
Treatment lines	
1	6 (37.5%)
2	8 (50%)
3	1 (6.25%)
More than 3	1 (6.25%)
Number of patients who received treatment lines	
First Line	15 (90%)
ITK inhibitor-sunitinib	15 (100%)
Immunotherapy	0
Second line	8 (50%)
Immunotherapy/nivolumab	5 (62.5%)
ITK inhibitor	3 (37.5%)
Axitinib	1 (12.5%)
Pazopanib	2 (25%)
Third line	3 (18.5%)
ITK inhibitor-axitinib	1 (33%)
Everolimus	1 (33%)
Immunotherapy/nivolumab	1 (33%)
Fourth line	1 (6.25%)
ITK inhibitor-axitinib	1 (100%(
Local treatment of disease in the central nervous system	
Radiotherapy	5 (31.5%)
Brain stereotaxic radiotherapy	1 (6.25%)
Surgery	1 (6.25%)

Around 68% of the patients underwent nephrectomy as local management of their primary disease. The distribution according to the risk measured by IMDC for the present study was 12.5% favorable, 43.7% intermediate, 25% poor, and 18.8% not classified.

Around 50% had a single lesion in the CNS at their initial neuroimaging, local treatment of the disease in the CNS was performed in seven patients, of these, palliative holocranial radiotherapies were offered in five patients and of the remaining two, one was managed with SBRT and the other with surgical management by neurosurgery of the lesion. Locoregional therapies in central nervous system disease were not offered simultaneously (e.g. radiosurgery and neurosurgery) and were required only at the progression to the first and second line of treatment. 

Of the total database of 16 patients, 90% (15) started first-line treatment and received sunitinib (a tyrosine kinase inhibitor). The patient who did not receive treatment was a patient who debuted with metastasis in the central nervous system and was thus a candidate for support management.

In the first line of treatment with sunitinib, 15 patients received an average of 23 months of treatment (SD of 23 months with a minimum of 0 months and a maximum of 70 months) and toxicity of 63% (n=9) occurred. The main toxicity was anemia in 55.5% (n=5), This toxicity according to the toxicity classification was grade 1 in four patients and grade 2 in one patient, followed by cardiovascular toxicity with grade 2 arterial hypertension in one patient, and grade 1 asthenia in one patient.

The main grade 4 drug toxicity was thrombocytopenia in a single patient. It was considered that the main cause for changing the line of treatment was the progression of the disease, not the toxicity associated with the treatment. In the first line of treatment of the four patients who debuted with CNS disease, three received local regional management, one patient received SBRT, one was treated with surgery, and one with holocranial radiotherapy.

The second line of treatment was started by 8 (50%) patients, and of these seven patients who presented with progression to the central nervous system, four (50%) patients received local treatment of the disease in the CNS, two patients were treated with SBRT and two with holocranial radiotherapy. Of the eight patients who reached first-line progression and received systemic treatment, five (62%) were treated with nivolumab; the mean duration of treatment was 11 months (SD 8.1 months with a minimum time of one and maximum of 24 months. The toxicity presented with nivolumab was hypothyroidism in two patients (40%) classified according to guidelines as grade I toxicity, not limiting treatment, and the cause of line change was the progression of the disease. The other three patients who progressed to the first-line TKI received a second TKI; additionally, two received pazopanib, and one received axitinib. Second-line TKI toxicity was 33% of the group, being grade 1 anemia toxicity for axitinib without being a cause for treatment change; the mean TKI treatment duration was 17 months (SD 17, 3 with a minimum of six and a maximum of 42 months).

The two patients who received local therapy and progressed to the second line are patients who are alive at the time of completion of the study observation. The third line of treatment was started by three patients and all were progressions to the central nervous system; none of these patients received local therapy and one patient received everolimus for 19 months, with no reports of toxicity. Another patient who received axitinib did not report toxicity for eight months.

The last patient received immunotherapy with nivolumab monotherapy for 10 months with no reported toxicity and the reason for discontinuation was a change of line due to progression. One patient reached the fourth line of treatment, had progression to the CNS in this line, received axitinib, the duration of treatment was less than one month, had no toxicity, and no locoregional management was reported; the reason for receiving the therapy for a short time period was the mortality associated with their underlying disease.

**Figure 1:**
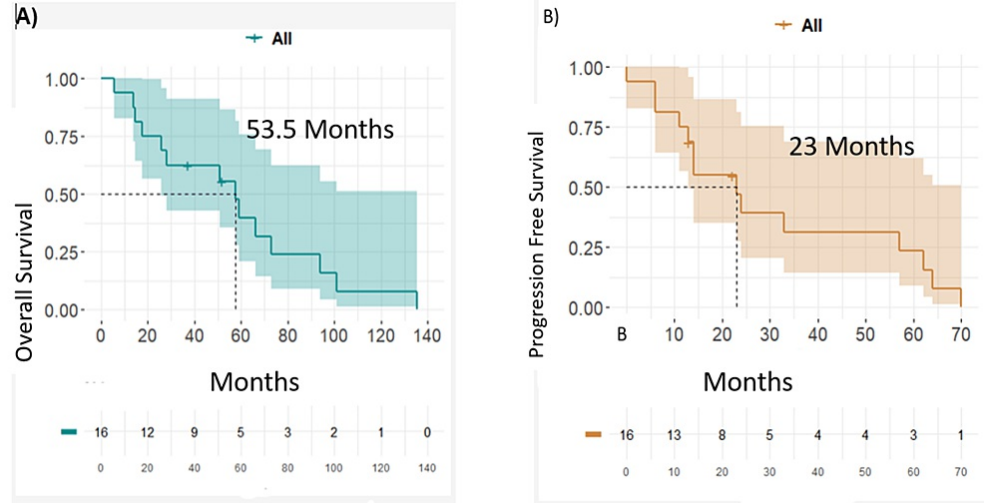
Overall survival and progression-free survival (A) Overall survival of the group counted from the time of diagnosis of metastatic cancer until death from any cause, including patients whose disease had not metastasized to the central nervous system. (B) Progression-free survival of the general population of the study.

The median follow-up time for the general group was 35.1 months (IQR: 44.1), which ranged from 5.85 to 135.4 months. Death had occurred in 14/16 patients (87.5%). Figure 1 presents the Kaplan-Meier curves for overall survival (OS) and progression-free survival (PFS), respectively.

**Figure 2:**
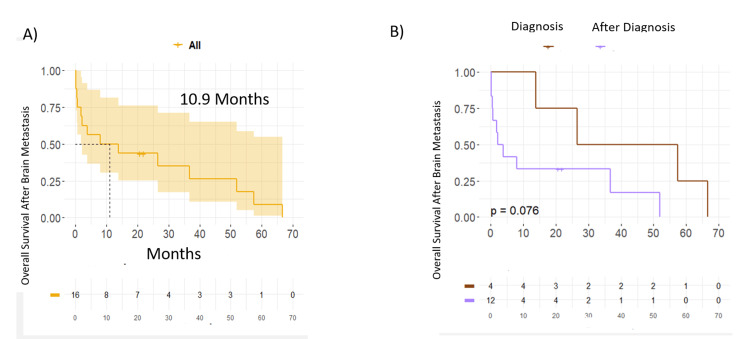
Overall survival at the time of diagnosis and after diagnosis of CNS involvement (A) Overall survival from the time of diagnosis of CNS involvement and death from any cause. (B) Overall survival at the time of diagnosis and after diagnosis of CNS involvement. CNS: central nervous system

The median PFS was 23.0 months and the median OS was 53.5 months. When stratifying by the IMDC status, there is no significant difference for the subgroups. The overall survival from the diagnosis of disease in the central nervous system and death is 10.9 months for the general population, leaving an overall survival of 42 months for the group that debuted with CNS involvement and an OS of 3.6 for the group that presented CNS involvement during the progression of their disease (Figure 2).

## Discussion

At the moment there is no evidence from phase 3 studies to determine the best treatment for mRCC patients with involvement of the central nervous system. 

Studies describing first-line metastatic disease have specifically excluded this population as they were considered high-risk with an unknown profile of biological behavior [5-8].

For the behavior of later lines, specifically in cases with compromised central nervous systems, there is only the GETUG-AFU 26 NIVOREN study, which showed that the compromise at the level of the central nervous system had an improvement when there were locoregional interventions, where 73 patients with documented BrM were divided into two arms; a cohort of 39 patients who did not receive local therapy, and another cohort of 34 patients who received local management, yielding a response rate 12% of the first cohort. This emphasizes the importance of local therapy before systemic therapy [9], unlike our study, where the most frequent measure once brain metastasis is diagnosed is systemic management without local treatment: only seven of the 16 patients received local management. 

Kotecha et al. published in 2021 an analysis of 68 phase 2 and 3 trials of patients with mRCCs who had to undergo brain screening at the time of recruitment; 72 patients were diagnosed with brain metastases [10], finding a multifocal brain involvement of 38.5% compared to 50% in our study. Close to 93% of the patients received local treatment, which was higher compared to the 40% of patients in our study who received treatment. 

The median overall survival for the study by Kotecha et al. is 10.3 months and 10.9 months for our study, independent of the time of diagnosis of central nervous system involvement, and like several studies, independent of the risk given by the IMDC [10-13], which does not seem to be a differential at the time of overall survival and central nervous system involvement. It is striking, but it is not possible to give any type of analysis other than descriptive, that overall survival in both studies was similar with differential local measures, raising a question as to whether local management is a prognostic variable in the treatment of this subgroup of patients. It should be noted that, in general, in the study by Kotecha et al., the patients were not only on clinical protocols but all the patients were asymptomatic compared to our cohort, which was generally a completely symptomatic population at the time of neuroimaging.

It is estimated that metastasis in the central nervous system at the time of the debut of renal metastatic disease is about 1-2%, however, in our study, it was 25% (n:4) [10,11]. While the lesion size has previously been associated with symptoms, lesion number has been associated with lower OS [12]. In our study, this analysis was not performed.

The time of diagnosis of the metastatic disease in the central nervous system was not taken into account for the calculation of overall survival, as was done in the study by Ratnayake et al. [13] where almost 60% of the patients were diagnosed in during the first line of treatment, giving an overall survival of 21 months. However, in our study the disease was not such a frequent manifestation in the first line (25%) but rather mostly manifested between the second and third lines; all the patients in the first line were candidates for systemic management; those who experienced CNS involvement were treated with immunotherapy monotherapy. 

There is no agreement in the guidelines for asymptomatic brain screening of patients with renal tumors, therefore it is difficult to establish the optimal time for screening and follow-up in the asymptomatic population [14-15].

In other studies, such as the Checkmate 920 trial, the median progression-free survival was 9.0 months (95% CI, 2.9-12.0 months), and median overall survival was not reached (95% CI, 14.1 months to not estimable) in the first line. Even without reaching mature overall survival, our study describes a survival with a lower limit of 14.1 months, which is higher than that described in other case series. Due to the current type of study, it is only possible to describe the behavior of the disease; therefore it is for future studies to determine if treatment in earlier lines could improve the general prognosis of the disease [16]. 

The main limitations of our study are due to the type of study (retrospective) with the usual drawbacks associated with this design. It is important to highlight that despite the differences between the type of patients in previous studies and our study, the biological behavior of the disease is similar, being aggressive and apparently independent of the classic risk variables. Therefore, it seems to be related to a different and more aggressive disease phenotype.

## Conclusions

Metastatic renal cell carcinoma in the nervous system is a spectrum of disease more aggressive that is not usually taken into account in conventional risk scores such as IMDC or Memorial Sloan-Kettering Cancer Center risk models; one possibility to consider is that central nervous system metastases and renal cell carcinoma could correspond to a different, more lethal disease phenotype. Therefore, these patients should be treated with the aid of a multidisciplinary team and have their choice of therapy.

## References

[1] Sung H, Ferlay J, Siegel RL, Laversanne M, Soerjomataram I, Jemal A, Bray F (2021). Global Cancer Statistics 2020: GLOBOCAN estimates of incidence and mortality worldwide for 36 cancers in 185 countries. CA Cancer J Clin.

[2] Abdel-Rahman O (2018). Impact of histological subtype on outcomes of renal cell carcinoma patients. J Drug Assess.

[3] Schouten LJ, Rutten J, Huveneers HA, Twijnstra A (2002). Incidence of brain metastases in a cohort of patients with carcinoma of the breast, colon, kidney, and lung and melanoma. Cancer.

[4] Bowman IA, Le T, Christie A (2016). Incidence of brain metastases in metastatic renal cell carcinoma in the era of targeted therapies. J Clin Oncol.

[5] Motzer RJ, Tannir NM, McDermott DF (2018). Nivolumab plus ipilimumab versus sunitinib in advanced renal-cell carcinoma. N Engl J Med.

[6] Parmar A, Chan KK (2020). Health technology assessment methodology in metastatic renal cell carcinoma. Nat Rev Urol.

[7] Motzer RJ, Penkov K, Haanen J (2019). Avelumab plus axitinib versus sunitinib for advanced renal-cell carcinoma. N Engl J Med.

[8] Choueiri TK, Powles T, Burotto M (2021). Nivolumab plus cabozantinib versus sunitinib for advanced renal-cell carcinoma. N Engl J Med.

[9] Flippot R, Dalban C, Laguerre B (2019). Safety and efficacy of nivolumab in brain metastases from renal cell carcinoma: results of the GETUG-AFU 26 NIVOREN multicenter phase II study. J Clin Oncol.

[10] Kotecha RR, Flippot R, Nortman T (2021). Prognosis of incidental brain metastases in patients with advanced renal cell carcinoma. J Natl Compr Canc Netw.

[11] Sun M, De Velasco G, Brastianos PK (2019). The development of brain metastases in patients with renal cell carcinoma: epidemiologic trends, survival, and clinical risk factors using a population-based cohort. Eur Urol Focus.

[12] Ferrel EA, Roehrig AT, Kaya EA (2016). Retrospective study of metastatic melanoma and renal cell carcinoma to the brain with multivariate analysis of prognostic pre-treatment clinical factors. Int J Mol Sci.

[13] Ratnayake G, Challapalli A, McGrane K (2022). A UK multicentre retrospective review of metastatic renal cell carcinoma (mRCC) patients (pts) outcomes with brain metastases (BM) in the modern era. Ann Oncol.

[14] Motzer RJ, Jonasch E, Agarwal N (2012). Motzer RJ , Jonasch E , Agarwal N , et al.. NCCN Clinical Practice Guidelines in Oncology: Kidney Cancer, Version 2.2020. J Natl Compr Canc Netw.

[15] Ljungberg B, Albiges L, Abu-Ghanem Y (2019). European Association of urology guidelines on renal cell carcinoma: the 2019 update. Eur Urol.

[16] Emamekhoo H, Olsen MR, Carthon BC (2022). Safety and efficacy of nivolumab plus ipilimumab in patients with advanced renal cell carcinoma with brain metastases: CheckMate 920. Cancer.

